# Plant bioclimatic models in climate change research

**DOI:** 10.1186/s40529-015-0104-8

**Published:** 2015-09-21

**Authors:** Chyi-Rong Chiou, Tung-Yu Hsieh, Chang-Chi Chien

**Affiliations:** 1 School of Forestry and Resource Conservation, National Taiwan University, No. 1, Sec. 4, Roosevelt Rd., Taipei, 10617 Taiwan (R.O.C.); 2 Shanghai Institutes for Biological Sciences, Chinese Academy of Sciences, 320 Yue Yang Rd., Shanghai, 200031 China; 3 Shanghai Chenshan Plant Science Research Center, Chinese Academy of Sciences, 3888 Chenhua Road, Songjiang, Shanghai, 201602 China; 4 Shanghai Key Laboratory of Plant Functional Genomics and Resources, Shanghai Chenshan Botanical Garden, 3888 Chenhua Road, Songjiang, Shanghai, 201602 China; 5 College of Business, Chung Yuan Christian University, 200, Chung Pei Rd., Chung Li, 32023 Taiwan (R.O.C.)

**Keywords:** Climate change, Phenological model, Theoretical model, Statistical model, Mechanistic model

## Abstract

Bioclimatics is an ancient science that was once neglected by many ecologists. However, as climate changes have attracted increasing attention, scientists have reevaluated the relevance of bioclimatology and it has thus become essential for exploring climate changes. Because of the rapidly growing importance of bioclimatic models in climate change studies, we evaluated factors that influence plant bioclimatology, constructed and developed bioclimatic models, and assessed the precautionary effects of the application of the models. The findings obtained by sequentially reviewing the development history and importance of bioclimatic models in climate change studies can be used to enhance the knowledge of bioclimatic models and strengthen their ability to apply them. Consequently, bioclimatic models can be used as a powerful tool and reference in decision-making responses to future climate changes. The objectives of this study were to (1) understand how climatic factors affect plants; (2) describe the sources, construction principles, and development of early plant bioclimatic models (PBMs); and (3) summarize the recent applications of PBMs in climate change research.

## Background

Bioclimatology or *bioclimatics*, which includes phenology, is an ancient science that investigates the relationship between living organisms and climates. According to historical records, China was the first country to conduct bioclimatic observation approximately 3,000 years ago. Bioclimatology is referred to as *Wuhou* (物候) in Chinese, a word that originated from the classic *Ch’un*-*ch’iu Tso Chuan* (春秋左傳). Western bioclimatology was established in approximately 1753 by Linnaeus, a Swedish botanist, who is known as the father of phenology. The term phenology was first introduced by the Belgian botanist Morren in 1853. One hundred years before the term was coined during Linnaeus’ time, phenology was focused on the seasonal and periodic phenomena that organisms exhibit and is referred to as classic or seasonal bioclimatology. In Japan, phenology is referred to as the study of seasons and organisms. Scientists have since identified that changes in living organisms follow periodic changes in climates. Thus, the scope and definition of phenology vary constantly as new bioclimatic findings are obtained. Consequently, the early definition of phenology has become inapplicable. Although numerous scientists have attempted to redefine phenology and create linguistically specific technical terms, many people prefer to use the established term phenology, which has been used continuously since it was coined. Bioclimatology, including phenology, now involves investigations of the correlations between climates and organisms (Chu and Wan 1999; Hopkins 1938; Hsieh and Chiou 2013; Lieth 1974; Schnelle 1955; Zou 1983). To avoid confusion caused by different definitions, this article defines all types of model that have both biological and climatic variables as bioclimatic models.

Despite its ancient origin, bioclimatology has long been disregarded because of problems, such as difficulty in funding long-term research in the past. In recent years, bioclimatology has received increasing attention and has become critical for investigating the effects of climate changes on organisms (Hänninen and Tanino 2011; Hsieh and Chiou 2013; Körner and Basler 2010; Lechowicz and Koike 1995). Initially, ancient people developed bioclimatology by recording the correlations between biological phenomena according to annual observations made during farming seasons and related experiences; in this way, lunar calendars and bioclimatic calendars were compiled. Thus, bioclimatic research development in ancient times was focused on agricultural phenomena and various biological indicators recorded in the bioclimatic calendars of different cultures were used as a disaster-prevention system for decision-making. Bioclimatology in the Western scientific field did not become a formal discipline until the mid-eighteenth century when Linnaeus established the first phenology observation networks in Sweden and emphasized the tasks and importance of phenological observations in his book *Philosophia Botanica* (Hsieh and Chiou 2013; Lieth 1974).

Because the threat of climate change has recently attracted increasing attention, phenology network records have been developed into two complementary research systems; one is the concept of bioclimatic fingerprints, which was developed from phenology observation networks and is used for observing and monitoring the effects of climate changes on organisms, and the other is bioclimatic modeling based on long-term bioclimatic records and variations of the phenology observation networks for clarifying the correlation between climates and organisms and predicting the possible effects of climate changes on organisms. The results can be used as references in future disaster alert systems, disaster-prevention decision-making, and the assessment of disaster effects (Peñuelas and Filella 2001).

Although bioclimatic models are essential to researching climate change effects and despite the rapid international development and application of bioclimatic models, research and reports regarding the application and exploration of bioclimatic models remain scant in many undeveloped and developing countries, which are severely threatened by climate change. To improve the capability of people to address the threat of climate changes, we reviewed the factors that influence plant bioclimatology, the construction and development of bioclimatic models, and the application of bioclimatic models in disaster prevention and impact assessment. The sequential review of the development history and importance of bioclimatic models in climate change research provided in this study can be used as references by researchers studying climate changes.

## Climatic factors that affect plant growth and development

Bioclimatic models represent the phenomena, processes, or mechanisms of the effect of climate factors on organisms. Thus, before understanding the modeling principles of bioclimatic models, basic knowledge regarding the environmental factors that affect plant bioclimatology must be acquired. The effects of environmental factors on plants vary with plant species, phenological phases, geographical environments, physiological statuses, and levels and types of ecological systems, yielding complex mechanisms. Among numerous environmental factors, temperature, water availability, and air flow (i.e., wind) are more closely related to climate changes and substantially affect plants.

### Temperature

The climatic conditions of different seasons and regions cause varying effects on the bioclimatology of different plants (Menzel et al. 2001). For example, the higher winter temperatures at middle latitudes cause most plants to blossom and sprout earlier (Sparkes et al. 1997). At middle and high latitudes, the end of growth periods and the beginning of dormant periods of most plants are primarily influenced by the shorter days and temperature conditions of late summer (Heide 1974; Wareing 1956). Subsequently, the low temperature of the following winter breaks plant dormancy (Fuchigami et al. 1982; Perry 1971; Vegis 1964). Fluctuating temperatures break plant dormancy more effectively than constant temperatures do (Campbell and Sugano 1975; Hänninen 1990; Murray et al. 1989). However for some plants, fluctuating and constant temperatures have the same effect (Myking 1997). Phenological variations during plant growth periods are primarily affected by accumulated temperature (Peñuelas and Filella 2001). However, selecting the initial temperature for calculating accumulated temperature has been a major difficulty in bioclimatology because it may differ substantially in plants of the same species when influenced by varying environmental factors (Heide 1993; Murray et al. 1989). This difference severely affects the precision and prediction accuracy in research regarding plant growth bioclimatology. Despite the differences, 5 °C is commonly used as the initial temperature for calculating the accumulated temperature of plants (Cannell and Smith 1986; Cannell et al. 1985; Kellomäki et al. 1995; Murray et al. 1989).

#### Water availability

In addition to temperature, water availability critically affects plant bioclimatology and is highly relevant to climate changes. However, the effects of water availability vary with species and other environmental conditions. In particular, the photoperiods and temperature conditions in tropical zones are relatively stable and variation in water availability is often the main factor influencing plant bioclimatology (Tissue and Wright 1995). For example, when it rains in tropical arid or semiarid climates, various plants blossom simultaneously, exhibiting high phenological synchrony (de Lampe et al. 1992). The following rainfall continuance affects the fruits of plants. A majority of tropical plants bear fruit in rainy seasons and the fruiting period is shortened or prolonged based on the precipitation of the current season (Bawa and Hadley 1991). Water shortage causes growth arrest among numerous plants, resulting in eco-dormancy (Reich and Borchert 1984). In high mountains and middle- to high-latitude areas, water availability and temperature changes resulting from thawing snow are key to plant blossom and growth (Walker et al. 1995).

#### Airflow

Airflow is also a critical climatic factor that affects plants. When daylight is sufficient, adequate airflow, such as a breeze or zephyr, facilitates the airflow exchange of leaves and promotes transpiration lowering the leaf and environmental temperatures. Airflow also assists the pollination of anemophilous plants; however, when the wind speed is excessively high, the photosynthesis of leaves is subdued; the stigmata of flowering plants dry up, which affects pollination and causes infertility; or soil drying and wind erosion are expedited, which results in exposed plant roots, fallen fruits, leaves, and flowers, and even severe mechanical injuries, such as broken and fallen stems. Consequently, trees are weakened because of malnutrition, diseases, pests, or infections, which cause alternate bearing, and eventually die from nutrition depletion (Campbell-Clause 1998; Duryea et al. 1996; Telewski 1995).

### Climatic factors in different growth and development stages

The effects of climatic factors on plants differ according to the various growth and development stages of plants. For example, climate conditions influence germination so that the germinating of seeds of different species and in various regions differs substantially. The seeds of plants that grow in temperate latitudes require low temperatures or fluctuating temperature conditions that last for a certain amount of time to break dormancy (Hsieh et al. 2004). However, numerous studies have shown that some temperate plant species can break seed dormancy through exposure to high temperatures and long photoperiod days (Isikawa 1954; Johnson and Irgens-Moller 1964; Stearns and Olson 1958). Some temperate species can break dormancy and sprout only after exposure to a period of low temperature following exposure to high temperature, such as *Taxus sumatrana* (Miq.) deLaub. (Chien et al. 1995) and peony seeds. The germination of seeds from numerous species also varies with environmental conditions, such as those for *Tsuga canadensis* L. The seeds of *Tsuga canadensis* L. break dormancy and germinate after exposure to 10 weeks of low temperature. However, the temperature required for germination of seeds that have not been exposed to low-temperature stratification increases with the length of photoperiod. For a general photoperiod of 8–12 h, the optimal germination temperature ranges from 17 to 22 °C, Whereas if the length of the photoperiod is 16 h, the optimal germination temperature increases to 27 °C (Stearns and Olson 1958). However, *Pseudotsuga menziesii* (Mirb.) Franco seeds that have not undergone low-temperature stratification can successfully germinate after a short-photoperiod below 25 °C (Johnson and Irgens-Moller 1964). The climate requirements and resistance may differ even among the various organs of a plant species. A survey exploring the freeze injuries of *Pyrus koehnei* C.K. Schneid. showed that 90 % of 6-year-old plants were frozen to death under −14 °C and 50 % of suckers were frozen to death under −12 °C, whereas only 28 % of stem surfaces exhibited freeze injury. The median lethal temperature of the different tissues ranged from −10 to −15 °C (Nee et al. 1995).

### Climatic factors in different areas

Climate changes may exert differing effects on the same species of plant in different areas with identical climatic conditions. For example, in certain areas of the former Soviet Union where the climatic conditions are identical, walnuts trees are frozen to death in autumn in certain locations but survive autumn in other places. A subsequent finding indicated that the difference is caused by varying photoperiods. In certain areas, the photoperiods shorten before the autumn frost, resulting in the early dormancy of walnuts. In other areas, the photoperiods are not short enough to induce bud dormancy. Therefore, with the same temperature during autumn frost, walnuts may be frozen to death in some areas but survive the frost in other areas (Haldane 1947). Photoperiods also influence the blossoming of strawberry flowers. Temperatures and photoperiods jointly regulate the differentiation of flower buds. Generally, long photoperiods imply that flower bud induction requires long durations at low temperatures whereas short photoperiods imply that flower bud induction requires short durations at low temperatures. Thus, in areas with the same temperature conditions, varying photoperiods may affect whether strawberry flowers blossom. The condition of the plant itself may also have an influence; for example, during flower bud induction, a decreased number of old leaves easily induces flower bud differentiation (Darnell and Hancock 1996).

Distinct microtopographies and microclimates influence precipitation; even a slight variation in rainfall may substantially affect plant growth. For example, at high altitudes, the fruiting amount of *Actinidia* is inversely proportional to the degree of overlap of flowering periods and the East Asian rainy season. A high degree of overlap implies low fruiting rates for a certain year, whereas a low degree of overlap increases the fruiting rate. Certain species lack fruit every year because the East Asian rainy season overlaps the flowering period. This severely affects the reproduction and growth of *Actinidia*. and damages economic growth related to the plants (Nee 1994). Moreover, plant species respond differently to climate changes. For example, if plants, such as *Acer saccharum* Marsh. and eastern hemlock, which originate from different regions, are planted at one location, the plants from the north areas or high altitudes stop growing early in the autumn (Nienstaedt and Olson 1961; Robak and Magnesen 1970). Altitudes also affect the temperature requirements and responses of plants. For example, the seeds and buds of *Actinidia* have different dormancy conditions at different altitudes. The higher the altitude, the higher the chilling requirement to break seed and bud dormancy (Fan and Nee 2007). By contrast, peach and cherry trees have lower chilling requirements at high altitudes (Huang 2011; Ou et al. 2000).

Based on the aforementioned research cases, we identified that understanding the physiological mechanisms through which climates affect plants is crucial to climate change research. The influence of climate changes on plants varies substantially with differences in species, region, and other influential factors. Therefore, if the physiological and ecological conditions of plants are not specifically controlled, constructing an appropriate bioclimatic model for climates with similar variable conditions and accurately evaluating and explaining the resulting influence of climate changes can be difficult.

## Bioclimatic model development

The origin of plant bioclimatic modeling is earlier than the formal establishment of bioclimatology. Such models can be traced back to 1735, when Reaumur proposed that the bioclimatic events of organisms and the dates of occurrence differ with regions, species, and altitude because the temperature required for each organism to grow and develop varies and accumulates differently according to region. This is the earliest degree-day summation concept, and for hundreds of years, this concept has been a fundamental basis for constructing bioclimatic models, such as the spring index model (Schwartz 1997; Schwartz and Marotz 1986, 1988), thermal time model (Cannell and Smith 1983; Robertson 1968), and spring warming model (Hunter and Lechowicz 1992).

After Reaumur, three types of bioclimatic models were developed in response to different research needs, methods, and objectives. Scientists refer to the three model types as theoretical, statistical, and mechanistic models. The theoretical model is also called the analytical model because it emphasizes the equilibrium between the productivity and the energy and nutrition absorption of leaves. Thus, because the model focuses on growth and development, it is suitable for research regarding the evolution of the survival strategies of species. The statistical model encompasses a wide and complex research scope. The primary objective of this model is to conduct statistical modeling, such as polynomial regression and general linear models, based on bioclimatic observation to directly connect climatic factors and biological events. Therefore, this model is also referred to as the empirical model. The mechanistic model focuses on the causal relationship between bioclimatic events and environmental factors to explain the effects of environmental factors on plant physiology. Because rigorous physiological and ecological theories and experimental bases support this model, its results are accepted relatively easily by a majority of scholars. The mechanistic model has been the standard of bioclimatic model research for a long period (Zhao et al. 2013). Except for the few bioclimatic models that use simple calculations, difficulties have typically been encountered during the early development of other bioclimatic models. These models were not developed and widely used until computer software and hardware became more easily accessible and a concomitant increase in the availability of data to parameterize such models (e.g., freely available gridded climate products) resulted in a stronger emphasis on global climate changes.

Each bioclimatic model has specific application restrictions and advantages and disadvantages. Scientists use the thermal time model most often because this model considers only the accumulated temperature, threshold temperature, and mean daily temperature of bioclimatic events as the parameters, facilitating model application. The model is shown as follows:

1 $$S_{f} = \sum\limits_{{t_{0} }}^{y} {R_{f} (X_{t} ) = F^{*} }$$

where S_f_ represents the accumulated units required to promote growth that satisfies bioclimatic event occurrence; $y$ represents the date of the bioclimatic event occurrence; $t_{0}$ represents the initial time for calculating the accumulated temperature; $X_{t}$ represents the mean daily temperature; and $R_{f} (X_{t} )$ represents the calculation function of effective accumulated temperature. This function is calculated using the following equation:

2 $$R_{f} (X_{t} ) = \left\{ {\begin{array}{*{20}l} {\begin{array}{*{20}l} 0 & \quad{\rm if} & {x_{t} \le T_{b1} } \\ \end{array} } \\ {\begin{array}{*{20}l} {x_{t} - T_{b1} } & \quad{\rm if} & x \\ \end{array} > T_{b1} } \\ \end{array} } \right.$$

where $T_{b1}$ represents the initial temperature for calculating the accumulated temperature. In this model, when the temperature is below the threshold growth temperature of a plant, the temperature does not influence phenological events. Only when the temperature exceeds the threshold growth temperature of a plant does the accumulated temperature affect phenological events. The higher the temperature, the greater the degree of influence is. However, this model is only applicable to the optimal temperature of plant growth. When the plant encounters extreme temperatures that exceed the optimal temperature of growth during the calculation of plant-accumulated temperature, the prediction errors of the model increase. Thus, several scientists have established the following formula to calculate the effective accumulated temperature based on the curves of plant growth development in response to temperatures.

3 $$R_{f} (x_{t} ) = \left\{ \begin{array}{ll} 0 & \quad {\rm if} \,\,\, {x_{t} < 0} \\ {\frac{a}{{1 + e^{{b(x_{t} - c)}} }}} & \quad {\rm if}\,\,\,{x_{t} \ge 0} \end{array}\right.$$

where c represents the optimal temperature for plant growth, b represents the parameter of plant sensitivity to variations in effective accumulated temperature, and a represents the upper limit of effective accumulated temperatures when bioclimatic events occur. This formula categorizes temperatures below 0 °C as noninfluential on bioclimatic events and involves only temperature accumulation above 0 °C.

The review of previous models shows that early thermal time models considered only the forcing units of growth, rather than the chilling requirements. In addition, during dormancy, plants are completely quiescent; thus, the phenological phase during dormancy is difficult to observe and define. However, a high number of physiological experiments in later stages have shown that low temperatures are necessary in winter for temperate plants to blossom and sprout. Bioclimatic models that neglect chilling requirements cannot effectively predict the flowering and sprouting of temperate plants. Therefore, scientists have developed numerous mechanistic models based on differing physiological plant types and have integrated chilling requirements into various models. Among these models, the most well-known are the sequential model (Hänninen 1987, 1990; Sanders 1975; Sarvas 1974), parallel model (Landsberg 1974; Sarvas 1974), alternating model (Cannell and Smith 1983; Kramer 1994; Murray et al. 1989), deepening rest model (Kobayashi et al. 1982), and four phase model (Hänninen 1990; Vegis 1964).

The differences between these bioclimatic models are as follows: The sequential model emphasizes that forcing temperature is effective only after chilling requirements are met, presenting a sequential order. Landsberg (1974) proposed the parallel model for identifying the dormancy characteristics of apple buds, indicating that regardless of temperatures, the phenological expression of plants is affected. The alternating model emphasizes that the forcing units and chilling units possess a negative indicative correlation. Thus, the two requirements alternatively influence phenological expression based on different weighting degrees with variations in the dormancy stages of plants. Kobayashi et al. (1982) proposed the deepening rest model in their study regarding the bud dormancy characteristics of *Cornus sericea* L. This model emphasizes that chilling requirements occur only during the deep rest stage, and that calculations of chilling requirements are not necessary for other dormancy stages. The four phase model emphasizes that plants have four sub-phenological phases during dormancy, which are the prerest, true-rest, postrest, and quiescence phases. The critical temperature-forced growth increases continuously during the prerest phase, but decreases during the postrest phase. In the true-rest phase, plants do not respond to any forcing growth temperature. The critical plant growth temperature decreases to the lower limit of initial temperatures for plant development in the postrest phase. When the external temperature remains below the lower limit temperature, plants enter the quiescence phase, the length of which is determined by the physiological conditions of the plant and the temperature increase in the following spring.

Regarding the measurement of the chilling requirements of plants in thermal time models, two common calculation methods exist:

4 $$R_{c} (x_{t} ) = \left\{ {\begin{array}{ll} 1 & \quad {\rm if} \,\,\, {x_{t} < T_{b2} } \\ 0 & \quad {\rm if} \,\,\, {x_{t} \ge T_{b2}}\end{array}} \right.$$

where $R_{f}$ becomes $R_{c}$, indicating that the growth accumulated temperature is replaced by the accumulated low temperature of chilling requirements, and $T_{b2}$ represents the upper limit of the critical temperature of effective low temperatures. Temperatures higher than $T_{b2}$ have no effect on the temperature accumulation of chilling requirements. Only temperatures lower than $T_{b2}$ affect the temperature accumulation of plant chilling requirements. Binary coding is adopted to calculate the effective accumulated temperature. In other words, regardless of temperature values lower than the critical temperature, one effective chilling unit is counted. Even if the temperature is −50 °C, which freezes plants to death, an effective chilling unit is counted. This formula obviously contradicts empirical experience. Therefore, subsequent scientists have developed another formula for calculating the effective chilling unit:

5 $$R_{c} (x_{t} ) = \left\{ \begin{array}{ll} 0 & \quad {\rm if}\,\,\, x_{t} \le T_{m}\,\,\,{\rm or}\,\,\,x_{t} \ge T_{M}\\ {\frac{{x_{t} - T_{m} }}{{T_{0} - T_{m} }}} & \quad {\rm if}\,\,\, T_{0} > x_{t} > T_{m}\\ {\frac{{x_{t} - T_{M} }}{{T_{0} - T_{M} }}} & \quad {\rm if}\,\,\, T_{0} < x_{t} < T_{M} \end{array}\right.$$

where $T_{m}$ and $T_{M}$ represent the upper and lower limits of the effective low temperatures of plants, respectively. When the external temperature is lower or higher than the upper and lower limits, the accumulated temperatures for plant chilling requirements are not effective. The term $T_{0}$ refers to the most effective chilling requirement temperature of plants. Clearly, this formula meets the actual situation more accurately than formula (4) does.

Different plant bioclimatic models combined with various plant physiological types must be calculated using different methods. For example, when thermal time models are used to predict plant flowering on the sequential model, the plant chilling requirements must be calculated and satisfied before the growth-accumulated temperature of plants is calculated. If parallel models are used, chilling accumulated temperature and forcing accumulated temperature must also be calculated to predict bioclimatic events. Hence, dozens of model combinations for predicting plant flowering or sprouting by using the thermal time model are available. The high degree of plant bioclimatic and physiological diversity contributes to the complex development of bioclimatic models. The complexity of bioclimatic model development, to a certain degree, effectively increases the accuracy of bioclimatic prediction; however, such complexity also impedes the promotion and application of the models. To simplify the application of bioclimatic models, Chuine (2000) combined numerous major mechanistic models and developed a set of unified bioclimatic model calculation methods, which comprises two formulas to calculate the forcing and chilling requirements of plants. Through the adjustment of various parameters in the model, Chuine fitted the plant differences resulting from physiological responses, phenological phases, regions, and latitudes. Subsequently, Chuine and Beaubien (2001) further argued that the distribution of woody plants is primarily determined by the degree of fitness of the plant bioclimatology to the local climates. Thus, they integrated other models, such as those of freeze injury and fruit ripening, to develop a bioclimatic model based on biological processes, which they referred to as the PHENOFIT model. The model uses bioclimatic observation data for parameter fitting of bioclimatic models and meteorological variable map layers provided by Environment Canada, Climate Archives, the National Climatic Data Center, and the World Radiation Center to determine species distribution according to the fitting degree of the species bioclimatology to the local climates. Because the PHENOFIT model combines multiple bioclimatic models, the calculation formula is complex. Nevertheless, the PHENOFIT model requires the input of only five variables to obtain 12 variables that explain the effects of climates on species. These resulting variables altogether can determine the distribution appropriateness of species. The PHENOFIT model uses climatic data from various geographic regions to infer the distribution of numerous temperate perennial woody plants. The results indicated that the outcomes inferred using the model highly corresponded to the actual distribution of the target species.

The temperature, light, water availability, and airflow changes caused by climate changes influence the transpiration rate of leaves, which is determined by numerous factors, such as the net radiation balance of leaves, water supply conditions, leaf shapes, environmental wind speed, and the reaction of the stomata to transpiration sensitivity (Gates 1968; Raschke 1960). The model is as follows:

6 $$S_{t} (1 - \alpha_{l} ) + L_{d} - \varepsilon \sigma T_{a}^{4} = \frac{{\rho C_{p} (T_{l} - T_{a} )}}{{r_{a} }} + \frac{{\rho C_{p} }}{{\gamma^{*} }}\frac{{(e_{o} - e_{a} )}}{{r_{s} + r_{a} }}$$

where $S_{t}$ represents the incoming solar radiation ( Wm^−2^); $\alpha_{l}$ is the albedo of the leaf; $L_{d}$ is the incoming longwave radiation (Wm^−2^); $\varepsilon \sigma T^{4}$ is the long-wave radiation emitted by the leaf at the leaf temperature ($T_{l}$); $\rho$ is the environmental air density around the leaf (kgm^−3^); $C_{p}$ is the specific heat of air (kPa); $T_{a}$ is the air temperature (°C); $r_{a}$ is the aerodynamic conductance to heat transfer (sm^−1^); $\gamma^{ * }$ is the psychrometric constant (kPa °C^−1^); $e_{0}$ is the saturated vapor pressure (kPa) at the current leaf temperature; $e_{a}$ is the actual vapor pressure (kPa); and $r_{s}$ represents the stomatal conductance (sm^−1^). Formula (6) shows that a slight change in the temperature affects multiple factors simultaneously. When the air temperature increases, the long-wave radiation absorption of leaves is affected, increasing the thermal load of leaves and changing the saturated vapor pressure in the atmosphere. Consequently, the actual vapor pressure is insufficient and causes the water transpiration rate of the leaf to increase along with water consumption. Thus, the model can effectively evaluate the effects of temperature, light, water availability, and airflow changes on plants according to climate changes. Moreover, stomatal conductance differs with the sensitivity of plant species and strains to climate changes (Hofstra and Hesketh 1969).

Because of article length limitations, we introduced only three major types of plant bioclimatic models. In addition to the models introduced in this study, other bioclimatic models are of importance in separate fields of development. Basically, the diversity of relationships between organisms and climates leads to diversity among statistical (empirical) models, such as the thermal time, degree-days, heat sums, growing degree-days, physiological time, and spring warming models. The physiological and genetic diversity of organisms contributes to the diversity of mechanistic models, such as the parallel, sequential, deepening rest, four phases, Utah (Richardson et al. 1974), positive chill (Linsley-Noakes et al. 1995), and North Carolina models (Gilreath and Buchanan 1981). The diversity of biological and statistical theories contributes to the diversity of theoretical models, such as the models based on carbon equilibrium, the interaction of hormones, survival and reproductive adaptation, ecological niches, genetic behaviors, biological processes, and remote sensing. Naturally, some of the models involve a certain degree of correlation, which occasionally enables their mutual and complementary combination.

By reviewing the development of early bioclimatic models, we identified the following tendencies: (a) The number of studies regarding the bioclimatic models for perennial species substantially exceeds that of those for annual plants. (b) The number of bioclimatic model studies on temperate plants is considerably higher than that of those on tropical and subtropical plants. (c) The number of bioclimatic model studies on woody plants is substantially higher than that of those on herbal plants. (d) The number of observational bioclimatic model studies is substantially higher than that of experimental studies. (e) The number of bioclimatic model studies on plants that sprout and blossom in spring is considerably higher than that of those on plants with different growth and development stages. (f) The number of bioclimatic model studies on crops greatly exceeds that of those on forest plants. The majority of the bioclimatic model research conducted after 1753 has focused on the flowering and sprouting models of temperate plants. Regarding other bioclimatic models, only a few model studies on fruit ripening bioclimatology were found (Piper et al. 1996; Song and Ou 1997). Moreover, research on the bioclimatic model of leaf colouring periods is scant (Chuine and Beaubien 2001).

## Application of plant bioclimatic models in evaluating the influence of climate changes

Plant bioclimatic models have been applied and developed in different fields, such as for predicting and evaluating the influence of climate changes on plant bioclimatology (Hänninen and Tanino 2011; Hänninen et al. 2007; Hao et al. 2001; Morin et al. 2009), improving the primary productivity of ecosystem (Kramer and Mohren 1996; Watsona et al. 2013), helping patients with pollinosis predict the time when pollen will occur in the air (Frenguelli and Bricchi 1998), assisting in crop or forest management and disaster-risk decision assessment, diagnosing the effects of climate on crop growth and development, predicting or assessing the correlations between species and their survival or adaptive strategy evolution (Chuine and Beaubien 2001; Morin et al. 2008), rebuilding regional climate environments in the past (Maurer et al. 2011; Menzel 2005; Yiou et al. 2012), forecasting the flowering time of cherry blossoms for developing the tourisy industry (Allen et al. 2014), and diagnosing the growth and development conditions of organisms as well as diseases and pests (Villalta et al. 2007). Unsurprisingly, these applications are correlated with one other to a certain degree. In recent years, plant bioclimatic models have been continuously applied to climate change research to evaluate the effects of climate changes on organisms. This implies that the importance of applying plant models in climate change-related research has constantly increased (Peñuelas and Filella 2001). Thus, this study introduced the application of bioclimatic models in assessing the influence of climate changes and in disaster prevention.

Initially, scientists focused on how plant sprouting and leaf expansion in the spring are correlated with freeze and cold injuries in the spring. Thus, statistical and mechanistic models regarding plant sprouting were the first models used to evaluate the effects of climate changes on plants. These models are often used to evaluate plants’ ability to resist freezing or frost injuries (Cannell 1985; Cannell and Smith 1986; Hänninen 1991) or the competition for light that occurs among different species after climate changes (Cesaraccio et al. 2004). As bioclimatic model research progresses, theoretical models such as the DORMPHOT model, which is based on theoretical processes, are frequently used to assess the effects and risks of extremely low temperatures and freezing and cold injuries on forests. Theoretical models are also used to assess the risks of native species being affected by climate changes (Kramer 1995; Kramer et al. 1996; O’Neill et al. 2010). Based on an empirical experiment, the DORMPHOT model was more accurate than traditional models in assessing tree sprouting (Caffarra et al. 2011; Zottele et al. 2011).

Regarding the assessment of the effects of climate changes on plant bioclimatology, productivity, vegetation structures, vegetation dynamics, and forest landscapes, forest gap models that contain climate variables are often used to explain the effects of climate changes on forest succession, growth, landscapes, and the structural variations of plant communities (Bugmann 2001; Keane et al. 2001; Prentice et al. 1993). Additionally, because of the differing sensitivities of the models, the response degree of forest primary productivity models varies with the model adopted (Leinonen and Kramer 2002; Vitasse et al. 2011). Common instances are the effects of energy and carbon dioxide flows on leaf expansion and falling leaf bioclimatology, and the model for assessing the relationship between leaf area index and seasonal evolution (Chase et al. 1996). In addition, empirical (statistical) degree-day growing models are frequently used in investigating the bioclimatic changes and carbon sequestration cycles in land surface models (Arora and Boer 2005; Baldocchi et al. 2005; Delpierre et al. 2009; Vitasse et al. 2011). Similarly, regarding the effects of climate changes on the carbon sequestration ability of vegetation, the large-scale biological sphere model based on forest ecological system processes, BIOME-BGC, includes information on leaf growth and falling dates as parameters and applies the information to three types of vegetation research (Running and Hunt 1993).

The prediction results of bioclimatic modeling or the models themselves can be integrated with other models with various purposes to conduct research on the effects of climate changes (Halofsky et al. 2013). For example, Bonan (1998) used the monthly leaf area indices predicted using the land surface model of the National Center for Atmospheric Research as model parameters and applied the parameters to the grids of the Community Climate Model to facilitate global climate change research. Kaduk and Heimann (1996) determined the precautionary and mechanical structures that identify bioclimatology phases in environmental conditions and applied the structure to land carbon cyclic model research. Botta et al. (2000) used remote sensing data to estimate leaf sprouting time and developed empirical prediction formulas to predict leaf bioclimatology dynamics and propose a global bioclimatology precautionary structure. In addition, other professional bioclimatic models of climate change for large-scale structures based on biospheres or ecological systems exist, such as the Frankfurt biosphere model established based on the carbon equilibrium structure; the Lund-Potsdam-Jena dynamic global vegetation model, which assesses ecological system dynamics, plant geography, and land field carbon cycles (Sitch et al. 2003); the Canadian Centre for Climate Modeling and Analysis integrated biosphere simulator model, which predicts leaf bioclimatology based on light and temperature functions (Foley et al. 1996); and the forest carbon model based on photosynthesis and transpiration (Chiang and Brown 2007). These models have been widely applied in large-scale climate change research in recent years.

Recently, ecologists have focused on the effects of climate changes on species distribution, the resulting habitat fragmentation, and relevant species conservation arguments (Channell and Lomolino 2000; Crimmins et al. 2013; Fan et al. 2013; Gavin et al. 2014; Pauli et al. 2014; Pimm et al. 2014; Renton et al. 2013). Thus, numerous species distribution models developed on the based of the climate ecological niche theory of bioclimatic models have been applied in research on the effects of climate changes on species distribution and habitats. A major portion of these models are also referred to as climate envelope models (CEMs) (Hijmans and Graham 2006), such as the maximum entropy models (Phillips et al. 2004), machine-learning-based artificial neural network models, and integrated species distribution models (e.g., BIOMOD) (Coetzee et al. 2009; Thuiller 2003). However, not all species distribution models are categorized as CEMs. For example, although the PHENOFIT model was developed on the basis of biological processes and many physiologically based SDMs (Kearney and Porter 2009) are used to evaluate the effects of climate changes on species distribution, they are not CEMs.

The mapped atmosphere-plant-soil system model (Lenihan et al. 2003, 2008) can be used to assess the effects of climate changes on vegetation distribution, ecological system productivity, or forest fires. Remote-sensing time sequential data can be used to measure and assess land field surface phenology for assessing the vegetation responses after fires (van Leeuwen et al. 2010). In addition, regarding large-scale biological effect research, the BIOME-BGC, CLASS, Interannual Flux Tower Upscaling Sensitivity Experiment, third generation Coupled Global Climate Model, I/O buffer information specification, Lund-Potsdam-Jena, National Center for Atmospheric Research Land Surface Model, and remote-sensing-based NDVI/NDWI models can be used for assessing the effects of climate changes on large areas of vegetation (Bonan 1998; Desai 2010; Foley et al. 1996; Sitch et al. 2003). These models are convenient for use in large plain areas; thus, they have been widely adopted by studies in numerous temperate continental countries in recent years.

The types, application methods, and purposes of bioclimatic models are numerous, and the predictive accuracy of the models is determined by (a) the quality and quantity of data, (b) whether the user selects and uses the most appropriate model, and (c) the accuracy in forecasting climate changes. Because scientists mostly focus on (a) and (b), this paper does not discuss item (c), which requires the expertise of meteorologists. In particular, the situation described in (a) is inevitable when any model is used. However, because various models require different levels of data sensitivity, the requirements for data quality and quantity also differ. The requirements for data quality and accuracy are strict and are often based on bioclimatic models driven by data, such as the maximum entropy model, CEMs, and machine-learning models used for species distribution modeling. Thus, the preparation and compilation works of data are critical in these types of model. Two conditions are used to determine whether a user has selected and used the appropriate model. The first condition is the user’s understanding of the target organisms’ physiology, ecology, behavior, or biology. For example, if the constrain conditions of the distribution of a species is not a climatic factor, using CEMs and current species distribution data to assess the effects of climate changes on species distribution may lead to considerable errors. Therefore, to use bioclimatic models to assess the effects of climate changes on organisms, is necessary to identify the period in the target organisms’ life cycle that is most sensitive to climate changes. Subsequently, based on the period, a suitable model should be selected for conducing assessment to maximize the effectiveness of the model. Choosing an inappropriate model to conduct assessment typically results in errors (Coetzee et al. 2009).

All applications of bioclimatic models in assessing the effects of climate changes have advantages and disadvantages (Elith et al. 2006; Hijmans and Graham 2006). For example, statistical models are the most widely used and are user-friendly and users are not required to consider biological processes, genetics, and physiology; however, they lack explanatory power for the research results and have a limited scopes of applications. Statistical models generally can not be applied to research on the effects on large areas of vegetation variations. Mechanistic models yield the highest explanatory power for the effects of climate changes, and thus have optimal assessment effectiveness. However, uncertainty of species’ physiological mechanisms is a constraining factor of using such models. For instance, users may be uncertain regarding what model to use to assess the effects of climate changes on the dormancy of *Sassafras randaiense* Hay. Rehder because the bud dormancy and physiology of the plant species have not yet been thoroughly investigated. Regarding the research on bioclimatic models for exploring the effects of climate changes, the successful application of models is determined by the user’s understanding of each model. Only by selecting suitable models can reliable assessment on the effects of climate changes be conducted and accurate results be attained.

In our previous review of climate change research on plants (Hsieh and Chiou 2013), we found that phenological gardens and phenological observation networks are used to record the effects of past climate changes on organisms in climate change research and monitor the direct influence of climate changes on organisms. Bioclimatic models are used to assess the possible effects of future climate changes and assist in making disaster-prevention decisions. Bioclimatic models and phenological observation networks are complementary in assessing the effects of climate changes; neither can be neglected. Without the historical records of phenological observation networks, bioclimatic models lack modeling data; without bioclimatic models, phenological observation networks lack the function of risk assessment and cannot assist in disaster-prevention decision-making. Thus, phenological fingerprints and models have been developed rapidly for applications in international climate change research. The use of regional phenological fingerprints, which was once a tool for small- to medium-scale spaces, has been expanded to continental and global scales through the establishment of global bioclimatic monitoring plans (Bruns et al. 2003; Parmesan and Yohe 2003; Root et al. 2003). Regarding the application of models, although the global bioclimatic models developed on the basis of remote sensing data have been widely applied in studies in temperate continental countries in Europe and North America, small- to medium-scale phenological fingerprints and models are more suitable for Taiwan because of its small terrain.

## Conclusion

The effects of global climate changes have increased in recent years. Numerous cities in Europe, the United States, China, and Japan were measured to have had high temperatures exceeding 40 °C for several consecutive days throughout the summer of 2013. Torrential rain has caused disasters in numerous regions and weather stations all over the planet measured atmospheric carbon dioxide concentrations exceeding 400 ppm, the highest in millions of years. Moreover, climate changes have exerted increasingly severe effects on plants and wildlife (Anderegg et al. 2012; Harley 2011; Ibáñez et al. 2008; Inouye 2008; Kaschner et al. 2011; Moritz et al. 2008; Rode et al. 2010; van Mantgem et al. 2009). These disasters indicate that the threats of climate change are ubiquitous. Because of the global impacts of disasters, we suggest that all countries’ government and relevant research units immediately establish international phenology gardens and network systems, develop phenological fingerprint observation technologies, improve the ability to monitor the effects of climate changes on global organisms, and employ long-term bioclimatic observation records to develop bioclimatic models that are suitable for local climates and disaster prevention. Consequently, the capacity for assessing the effects of climate changes and predicting and preventing disasters can be prepared, and measures and strategies can be prepared in response to disasters caused by climate changes.
